# Comparison of Perioperative Outcomes Between Precise and Routine Segmentectomy for Patients With Early-Stage Lung Cancer Presenting as Ground-Glass Opacities: A Propensity Score-Matched Study

**DOI:** 10.3389/fonc.2021.661821

**Published:** 2021-04-27

**Authors:** Xianning Wu, Tian Li, Chuankai Zhang, Gao Wu, Ran Xiong, Meiqing Xu, Dan Su, Mingran Xie

**Affiliations:** ^1^ Department of Thoracic Surgery, The First Affiliated Hospital of USTC, Division of Life Sciences and Medicine, University of Science and Technology of China, Hefei, China; ^2^ School of Nursing, Anhui Medical University, Hefei, China

**Keywords:** early-stage lung cancer, ground-glass opacities, segmentectomy, video-assisted thoracoscopy, propensity score

## Abstract

**Introduction:**

Segmentectomy is widely used for early-stage lung cancer presenting as single or multiple ground-glass opacities (GGOs). Precise segmentectomy is the recommended procedure in China. However, clinically, most routine segmentectomies are performed using only high-resolution computed tomography (CT). The aim of this study was to evaluate the effect of two segmentectomy approaches for GGOs in the lung.

**Methods:**

From January 2020 to September 2020, 55 precise segmentectomies performed with real-time guidance using 3D reconstruction and 343 routine segmentectomies for patients with single or multiple GGOs were performed as uniportal procedures. To reduce bias related to outcomes, preoperative clinical factors were used for propensity score matching (1:1); 55 precision and 55 routine segmentectomies were selected and further analyzed. Perioperative outcomes, namely operation time, blood loss, resection margins, number of removed lymph nodes, postoperative pulmonary function (1 month after surgery), length of postoperative stay, and postoperative complications were compared between the two groups.

**Results:**

Patients constituted 43 men and 67 women, with an age range of 25–68 years (median: 53 years). No significant differences were seen between the groups regarding blood loss, complications, histological type, and postoperative pulmonary function, and there were no 30-day postoperative deaths in either group. The median operation time for the Precision group (74 min) was longer than in the Routine group (55 min) (*p <*0.01), and the number of removed lymph nodes in the Precision group (5 ± 1.1) was higher than in the Routine group (3 ± 0.8) (*p <*0.01). Chest tube duration days and postoperative stay days were similar in both groups; however, the rate of air leakage on postoperative day 1 was higher in the Precision group (*p* = 0.020). All patients in the Precision group had adequate resection margins. Four patients (7.3%) undergoing complex segmentectomy in the Routine group had inadequate resection margins and required resection of additional lung tissue.

**Conclusion:**

Routine segmentectomy can significantly shorten the operation time and might prevent postoperative air leakage in uniportal segmentectomy for lung GGOs. However, precision segmentectomy may be more precise for complex cases, ensuring adequate resection margins and lymph node dissection.

## Introduction

Owing to widespread computed tomography (CT) screening, very early-stage primary lung cancer appearing as ground-glass opacities (GGOs) is increasingly detected (1). The current standard surgical procedure for early-stage lung cancer (T1) is lobectomy, in accordance with the randomized controlled trial results of the Lung Cancer Study Group in 1995 (2). As GGO-featured lung adenocarcinoma is generally indolent, the previous standard procedure of lobectomy remains controversial. However, the recent Japan Clinical Oncology Group study (JCOG0804) results provide timely and important evidence supporting sublobar resection for small GGO-dominant lung adenocarcinomas (3).

Segmentectomy, first described in 1939 (4), is an important mode of sublobar lung resection and is widely used in patients with GGO-featured lung adenocarcinoma. Regarding tumor treatment, achieving sufficient resection margins is critical to prevent local recurrence (5–7). To ensure adequate surgical margins and to overcome the challenges of identifying nodules, multiple localization techniques have been proposed, including video-assisted thoracoscopic (VATS) segmentectomy. However, this procedure is challenging because of the requirement to understand the complicated anatomical variations of the segmental bronchi and vessels.

Precise segmentectomy is a relative concept derived from ‘precision surgery’, which has not been well defined previously. Precise segmentectomy means more accurate identifying of small variations, transecting of small target segmental bronchi and vessels, as well as preserving of small intersegmental vessels. These ‘small’ anatomical structures are difficult to identify on CT and may not be essential for routine anatomical segmentectomy. Precise segmentectomy has been strongly promoted with the development of new techniques, such as three-dimensional (3D) reconstruction, 3D printing, and electromagnetic navigation bronchoscopy. Without precise techniques, incorrect bronchial transection and vessel ligation would be questioned and argued, especially for live surgery and meeting presentation in China. However, most routine anatomical segmentectomies are performed using only high-resolution CT, and higher complication rates have not been observed. We hypothesized that routine segmentectomy for GGOs could achieve the same safety and accuracy of precise segmentectomy. The aim of this study was to evaluate the effect of precise segmentectomy using real-time 3D reconstruction navigation vs routine segmentectomy, for lung GGOs. Actually, clinical selective tendency exist in the real world, 3D reconstruction techniques would be selected more frequently when surgery live teaching or communicating, during which complex segmentectomies are more popular owing to high difficulty and educational value. Propensity score matching was used to reduce the selective bias in this retrospective study.

## Methods

### Patient Selection and Data Collection

We retrospectively analyzed 398 patients with single or multiple GGOs diagnosed as T1aN0M0 stage IA lung cancer at the First Affiliated Hospital of USTC from January 2020 to September 2020. Fifty-five precise segmentectomies guided by 3D reconstruction (Precision group) and 343 routine segmentectomies (Routine group) were performed as uniportal procedures. All protocols were reviewed and approved by the Ethics Committee of the First Affiliated Hospital of USTC. We retrospectively collected the following data from the medical databases: patients’ demographics; perioperative outcomes, namely operation time, blood loss, resection margin distance, number of dissected lymph nodes, postoperative pulmonary function (1 month after surgery), and complications; and length of postoperative stay.

**Figure 1:**
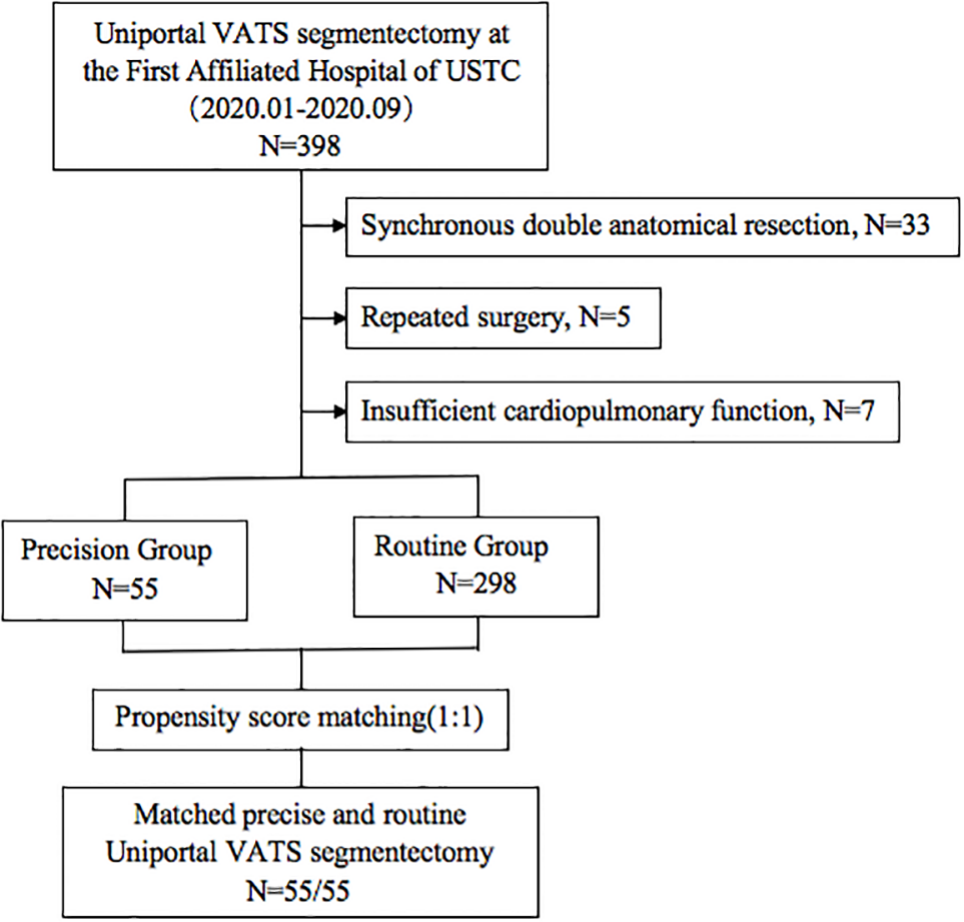
The flow chart of patient selection.

The indications for segmentectomy and preoperative evaluation were in accordance with the National Comprehensive Cancer Network (NCCN) guidelines, as follows: all enrolled patients with peripheral nodules <2 cm in size with at least one of the following were included: (1) pure adenocarcinoma in-situ, histologically; (2) nodules with 50% ground glass appearance on CT imaging; and (3) radiologic images confirming a long doubling time (>400 days) (8). Patients with insufficient cardiopulmonary function or other contraindications for segmentectomy were excluded. We limited the inclusion criteria to unilateral and single anatomical resection in each operation. Patients undergoing synchronous double anatomical resection, concurrent bilateral surgery, and repeated surgery in the same hemithorax were excluded (**Figure 1**).

Preoperative examinations constituted routine blood testing, pulmonary function testing, and high-resolution CT of the chest with or without contrast. Brain magnetic resonance imaging (MRI), bone scintigraphy, or flexible bronchoscopy were selected if necessary (e.g. for patients with complaints of dizziness, non-traumatic bone pain and irritable cough). Positron emission tomography, mediastinoscopy and endobronchial ultrasound-guided transbronchial needle aspiration (EBUS-TBNA) were not routinely performed on the basis of no swelling of mediastinal or hilar lymph nodes were revealed according to CT results. The pathological staging was in accordance with the criteria of the American Joint Committee on Cancer (AJCC) pathological tumor/node/metastasis (pTNM) classification (eighth edition). The pathological results of the GGOs were classified according to the 2011 International Association for the Study of Lung Cancer (IASLC), American Thoracic Society (ATS), and European Respiratory Society (ERS) (IASLC/ATS/ERS) classification (9). Postoperative complications were defined as grade ≥2 according to the Clavien–Dindo classification system (10).

### Preoperative Surgical Plan and 3D Reconstruction

The target segment and surgical plans were devised by surgeons with expert knowledge of the anatomic structures, and with correct and thorough interpretation of the CT images. All patients underwent preoperative CT with a slice thickness of 1.0 mm. Localization techniques were routinely used to mark the nodule, especially for GGOs located in basal segment or nearby intersegmental plane. For easily identified GGOs (e.g. subpleural nodule in relation to fissure) and simple segmentectomies with GGOs far from resection margin, localization techniques were waived (13/55 in Precision group, 14/55 in Routine group). The localization devices with small four-hook anchor and scaled suture were used as previously reported (11). The safe resection margin was defined as a sphere, extending 2 cm outside the primary tumor.

**Figure 2:**
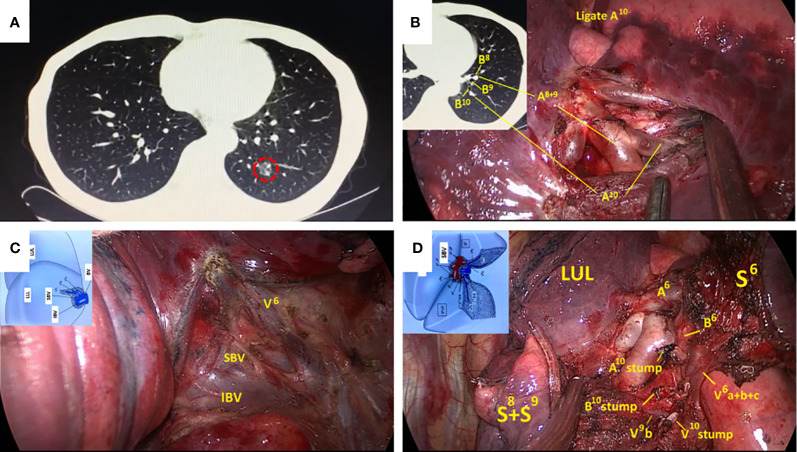
Routine left S^10^ segmentectomy based on CT scan and normal anatomic characteristics. **(A)** CT scan showed a GGOs lesion in the left S^10^; **(B)** The branches of the pulmonary artery exposed in the VATS were consistent with CT. **(C, D)** Surgical details were compared with segmental atlas written by Hiroaki Nomori and Morihito Okada. CT, computed tomography; VATS, video-assisted thoracoscopy; GGOs, ground-glass opacities.

**Figure 3:**
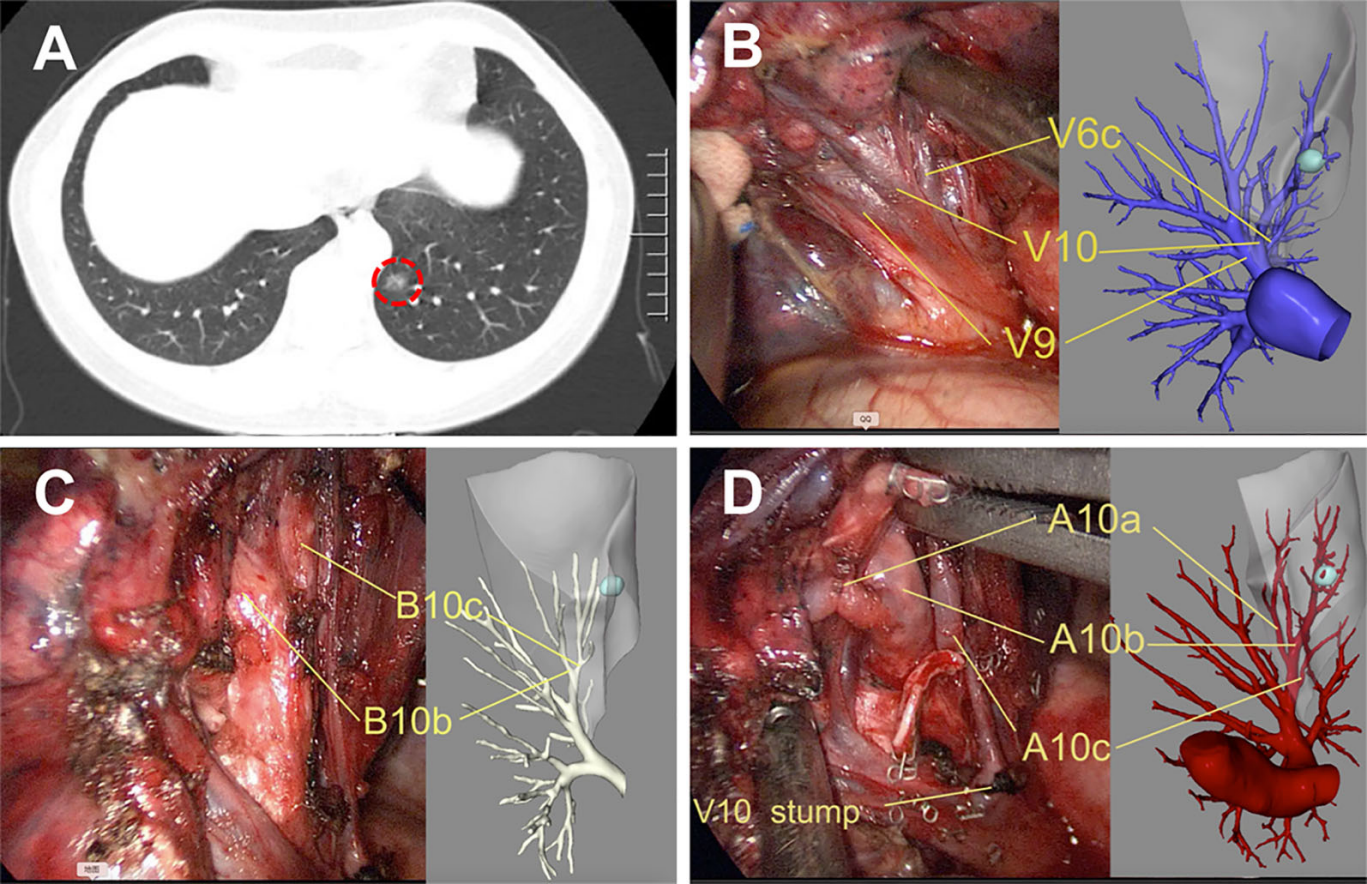
Precise left S^10^ segmentectomy according to the real-time 3D reconstruction guidance. **(A)** CT scan showed a GGOs lesion in the left S^10^; Branches pulmonary vein **(B)**, bronchus **(C)**, and pulmonary artery **(D)** of the target segment were confirmed by real-time guidance of 3D reconstruction. 3D, three-dimensional.

In the Routine group, the diagnostic and surgical plans were devised according to the serial CT images (**Figure 2**). Surgical margins were confirmed by intraoperative exploration and specimen frozen sections. Enlarged wedge resection was performed for insufficient margins. In the Precision group, contrast-enhanced CT was necessary, and digital imaging and communications in medicine (DICOM) data for each patient were recorded. 3D images were reconstructed using Inlook3D (INCOOL Tec., Wuhan, China). The validity of the reconstructions was confirmed, and images were preoperatively evaluated by the thoracic surgeons. All vessels and bronchi to the target segment were checked and marked on the 3D images, and safe resection margins and the relationships between the margins and the intersegmental veins were evaluated (12) (**Figure 3**).

### VATS Segmentectomy Procedure

Patients underwent surgery with general anesthesia with selective one-lung ventilation. Segmentectomy was performed in all patients *via* a uniportal approach with an incision of approximately 3–4 cm in the fourth or fifth intercostal space between the anterior and posterior axillary lines (13). Segmentectomy is defined as resection of the target segment after dividing the segmental pulmonary bronchus and artery, and selectively, the inter- and intrasegmental pulmonary veins. The intersegmental plane was demarcated using an inflation-deflation method (14). Intersegmental fissures and the bronchus were transected using a stapler, and stapling was also used to perform the segmentectomy to minimize air leakage from the lung parenchyma. Sealant tissue glue was routinely used. Sampling or dissection of segmental, lobar, hilar and mediastinal lymph nodes was performed when frozen section diagnosis of malignancy was performed.

In the Precision group, 3D reconstructions were available inside the operating room, and the segmental vein, artery, and bronchus were individually dissected according to the real-time 3D reconstruction guidance. The actual anatomy was compared with the 3D reconstructions, when there was any doubt. In the Routine group, the bronchus and main vessels were identified according to the CT images and the relationships between the blood vessel direction and the target segment. When thin vessels could not be identified, we attempted to preserve the vein and selectively dissect the artery.

### Propensity Score Matching (PSM)

The propensity scores were analyzed by logistic regression models to increase the sensitivity of the comparisons between the groups, and the matched factors were sex, body mass index (BMI), surgery type (complex vs simple), and the percentage of forced expiratory volume in 1 s (FEV1). Segmentectomy that created several, intricate intersegmental planes, with a more complicated procedure, was considered complex segmentectomy; that is, segmentectomy other than simple segmentectomy (15). Through the matching procedure for propensity scores, the Precision group and Routine group showed similar distributions of propensity scores, indicating that the differences in covariates between the two groups were minimized. Propensity scores were matched one by one using nearest neighbor matching methods, no replacement, caliper 0.025, and match 1:1.

### Statistical Analyses

Statistical analyses were performed using SPSS version 20.0 (IMB Corp., Armonk, NY). Normally distributed data are shown as the mean ± standard deviation. Mean values were compared using Student’s t-test, and frequency distributions were compared using the Chi-square test or Fischer’s exact test. A *p*-value of <0.05 was considered significant.

## Results

A total of 353 patients who underwent VATS segmentectomies were included in this study. 55 (15.6%) patients constituted the Precision group, and 298 (84.4%) patients constituted the Routine group. Before matching, there was a tendency toward more complex segmentectomies in the Precision group (*p* = 0.057). After matching, there were 43 male and 67 female patients, aged 25–68 years (median: 53 years). The matched groups were comparable regarding the patients’ baseline characteristics (**Table 1**), and the distribution of the complex segmentectomies is detailed in **Table 2**.

**Table 1:** Demographic data of precise and routine VATS uniportal segmentectomy.

Variables*	Before matching				After matching		
	All	Precision	Routine	*p* Value	Precision	Routine	*p* Value
	(N = 353)	(N = 55)	(N = 298)		(N = 55)	(N = 55)	
Age(years)	50.5 ± 5.3	52.9 ± 10.4	47.6 ± 12.6	0.108	52.9 ± 10.4	53.1 ± 11.9	0.978
Gender							
Male	162 (45.9)	20 (36.4)	142 (47.7)	0.142	20 (36.4)	23 (41.8)	0.696
Female	191 (54.1)	35 (63.6)	156 (52.3)		35 (63.6)	32 (58.2)	
BMI	23.6 ± 3.2	23.9 ± 3.6	23.5 ± 2.7	0.342	23.9 ± 3.6	23.8 ± 3.0	0.898
Surgery Type							
Simple	164 (46.5)	19 (34.5)	145 (48.7)	0.057	19 (34.5)	19 (34.5)	1.000
Complex	189 (53.5)	36 (65.5)	153 (51.3)		36 (65.5)	36 (65.5)	
Pre-op FEV1(L)	2.36 ± 0.42	2.38 ± 0.61	2.30 ± 0.38	0.406	2.38 ± 0.61	2.37 ± 0.74	0.933

*Variables used for estimating propensity score. BMI, body mass index; FEV1, forced expiratory volume in 1 s; VATS, video-assisted thoracoscopic surgery.

**Table 2:** Types of VATS segmentectomy in Precision group and Routine group.

Surgery types	Precision group	Routine group
Rright upper lobe	12 (21.8%)	13 (23.6%)
S^1^	2	2
S^2^	2	2
S^3^	3	3
S^2b + 3a^	5	6
Right lower lobe	14 (25.5%)	13 (23.6%)
S^6^	3	3
S^8^	3	3
S^9^	2	2
S^10^	5	5
S^9 + 10^	1	0
Left upper lobe	16 (29.1%)	16 (29.1%)
S^1 + 2(a + b)^	7	6
S^1 + 2(c)^	1	2
S^1 + 2^	3	3
S^3^	2	3
S^1 + 2 + 3^	1	1
S^4 + 5^	2	1
Left lower lobe	13 (23.6%)	13 (23.6%)
S^6^	2	2
S^8^	1	1
S^9^	3	2
S^10^	6	7
S^9 + 10^	1	1

Complex segmentectomy: Right S^2b + 3a,^ S^3^; left S^1 + 2(a + b)^, S^3^; and all S^8^, S^9^, S^10^ and S^9 + 10^.

There was no 30-day postoperative mortality in either group. Perioperative outcomes are compared in **Table 3**; no significant differences were seen between the groups for blood loss, histological type, complications, and postoperative pulmonary function. In the Precision group, pathological results identified four patients with adenocarcinoma, 41 with microinvasive adenocarcinoma (MIA), and 10 with adenocarcinoma *in situ* (AIS). In the Routine group, three patients had adenocarcinoma, 46 had MIA, and six had AIS, pathologically.

**Table 3:** Intraoperative and postoperative characters of Precision group and Routine group.

Variables	Precision group (N = 55)	Routine group (N = 55)	*p* Value
Operation time (min)	74 ± 14.6	55 ± 17.8	<0.01
Intraoperative blood loss (ml)	33 ± 7.5	28 ± 9.0	0.215
Inadequate resection margins	0	4 (7.3%)	0.118
Number of removed lymph nodes	5 ± 1.1	3 ± 0.8	<0.01
Histological type			0.108
Adenocarcinoma *in situ*	10 (18.2%)	3 (5.5%)	
Microinvasive adenocarcinoma	41 (74.5%)	46 (83.6%)	
Invasive adenocarcinoma	4 (7.3%)	6 (10.9%)	
Chest tube duration days	3.9 ± 1.7	3.7 ± 1.9	0.687
Air leakage on POD1	31 (56.4%)	19 (16.4%)	0.020
Postoperative hospital stay (days)	4.4 ± 1.3	4.1 ± 2.2	0.619
Postoperative complications			
Air leakage (>7 days)	2 (3.6%)	3 (5.5%)	
Pneumonia	0	1 (1.8%)	
Atrial fibrillation	1 (1.8%)	0	
Hemoptysis (>10 ml)	0	0	
Total	3 (5.5%)	4 (7.3%)	1.000
Post-op FEV1 (L)*	2.00 ± 0.53	1.89 ± 0.71	0.741

^*^Postoperative pulmonary functions were evaluated 1 month after surgery. POD, Postoperative Day. All patients’ air leakage on POD1 were under grade II.

The median operation time in the Precision group (74 min) was longer than that in the Routine group (55 min) (*p <*0.01). No lymph node metastasis was detected in any of the patients. Most of the examined lymph nodes were from stations 10 to 12, mediastinal lymph nodes were less and mainly from invasive adenocarcinoma cases. The number of dissected lymph nodes in the Precision group (5 ± 1.1) was higher than in the Routine group (3 ± 0.8) (*p <*0.01). Chest tube duration days and postoperative stay days were similar in both groups; however, the rate of air leakage on postoperative day 1 was higher in the Precision group (*p* = 0.020).

All cases in the Precision group had adequate resection margins (as defined in *Methods*). Four patients (7.3%) undergoing complex segmentectomy in the Routine group had inadequate resection margins (less than 2 cm) confirmed by intraoperative specimen evaluation, and additional wedge resection were performed. All resection margins were negative, identified by pathological analysis.

## Discussion

Excellent survival and low recurrence is associated with lung cancers involving GGOs (16). Therefore, it is reasonable to consider limited resection as an alternative to lobectomy for small, indolent lesions. Segmentectomy and wedge resection are the main sublobar resection procedures with the advantage of lung sparing. Results from JCOG0804 indicated that simpler procedure of wedge resection is suitable for GGOs with consolidation-to-tumor ratio ≤0.25. Whether segmentectomy could be the standard of care for other type GGOs, more randomized controlled trial results are needed. When choosing segmental lung resection, the oncologic curative effect should be verified first. Sufficient resection margins are critical to achieve a curative effect (5–7). If the expected oncologic outcomes are equivalent between procedures, the simpler procedure is preferred. In our study, routine segmentectomies were performed six times more often than precise segmentectomies during the same period. There are non-surgical reasons for this difference; precise segmentectomy takes more time because of the need to prepare the 3D reconstructions, and using 3D simulation services increases the medical costs. In comparison, for routine segmentectomy, non-enhanced CT is sufficient, and this method is more convenient and acceptable to patients. Additionally, clinical selective tendency existed in our clinical practice, 3D reconstruction techniques were selected more frequently for complex cases, because complex segmentectomies with high difficulty and educational value were more popular for surgery live teaching or communicating.

Segmentectomy is more complicated than lobectomy owing to the complex anatomy and variations in peripheral bronchi and vessels. In our study, the operation time in the Routine group was significantly shorter than that in the Precision group, which differs from findings in previous reports (17). The following reasons may explain this difference: For precise segmentectomy, 3D reconstruction can illustrate variations in anatomical details (18). To ensure accuracy and safety in precise segmentectomy, it is necessary to compare the actual anatomy with the 3D reconstructions repeatedly, especially for tiny vessels. Additional operation time may also be required for extended dissection of the lung tissue. In routine segmentectomy, which is based on normal patterns and individual CT data, the main anatomical characteristics are sufficient to achieve safe and quick segmentectomy, as confirmed by past clinical experience. With routine segmentectomy, thin vessels are not a concern, and unnecessary lung dissection is avoided. The incidence of complications in the two groups in this study was similar, and complications were rare. There was no thoracotomy-related or postoperative 30-day mortality in either group, which confirmed the safety of routine segmentectomy.

Despite extensive dissection in the Precision group, the chest tube duration days and postoperative stay days were similar in both groups. Moreover, we observed that the rate of air leakage on postoperative day 1 was higher in the Precision group. Air leakage is common with segmentectomy because of the dissection of the lung parenchyma (19). Most of the patients in this study had adequate lung function before surgery, indicating good lung compliance and elasticity. Additionally, using sealant tissue glue may be very helpful to prevent prolonged air leakage.

Ensuring adequate surgical margins is important with segmentectomy. In the Precision group in this study, all patients had adequate resection margins, which may have resulted from an accurate surgical plan; accurate vessel handling leads to clearer segmental boundaries showed by inflation–deflation method. Four patients undergoing complex segmentectomy in the Routine group had inadequate resection margins. In addition to unclear boundaries resulting from inappropriate transection of small vessels, the boundaries of simple segments are generally planar, whereas the boundaries of complex segments are often multiplanar (20). For planar unclear boundaries, intentional extended resection is easy, however, extended resection in angle area between unclear miltiplanar boundaries of complex segments is difficult to plan and perform, which may result in inadequate resection margins. Therefore, clear segmental boundaries obtained from precise segmentectomy are valuable for complex cases. Compared with inflation-deflation method, alternative techniques like indocyanine green injection may enhance the identification of the right intersegmental plane based on accurate vessel handling (21).

The guidance of 3D reconstructed images not only enables achieving safe surgical margins but also minimizes the anatomic resection of the lung tissues, which ensures oncological efficacy and retains more healthy tissue (18). However, no postoperative pulmonary function benefits were observed in this study because the pulmonary function of most of the enrolled patients was normal.

Generally, intentional lymph nodes sampling or dissection would be done for MIA and invasive adenocarcinoma respectively. In real world, we often harvest lymph nodes during bronchus and vessel dissection process. In the Precision group, segmental hilar structures were freed very well, which maybe the reason for the higher numbers of examined lymph nodes. Whether lymph nodes sampling or dissection is necessary for MIA patients remains controversy. This procedure may increase surgical injury. And excessive dissection may take more difficulties for possible second surgery in young patients. Further studies should work on this.

All of the patients in this study underwent intensive radical segmentectomy according to the NCCN guidelines for non-small-cell lung cancer (NSCLC), including seven patients diagnosed with adenocarcinoma with >5-mm invasive range. It is not uncommon that GGO-dominant lesions turn out to be invasive adenocarcinoma, pathologically (22). Whether segmentectomy is sufficient for invasive adenocarcinoma requires validation in future studies.

One of the limitations of this study was the retrospective design. Furthermore, although we used PSM to minimize baseline differences between the two groups, there were additional limitations. First, the sample size was small. Second, no long term follow-up results were included. Moreover, our results revealed an advantage of precision segmentectomy regarding ensuring adequate surgical margins in complex segmentectomy; however, the subgroup sample was too small to confirm this advantage between the two groups. Prospective studies with larger patient numbers are warranted to identify which surgical pattern is feasible and efficient for real-world segmentectomy to treat GGOs.

## Conclusion

Routine segmentectomy can significantly shorten the operation time and might prevent postoperative air leakage in uniportal segmentectomy for lung GGOs. However, precision segmentectomy may be more precise for complex cases, ensuring adequate resection margins and lymph node dissection.

## Data Availability Statement

The raw data supporting the conclusions of this article will be made available by the authors, without undue reservation.

## Ethics Statement

This study involving human participants was reviewed and approved by the Ethics Committee of the First Affiliated Hospital of the University of Science and Technology of China. Written informed consent for participation was not required for this study in accordance with our national legislation and the institutional requirements.

## Author Contributions

All listed authors have made a substantial, direct, and intellectual contribution to the work, and approved the manuscript for publication. XW and MrX contributed to the study design. TL, CZ, GW, and MqX were responsible for interpreting the results. DS, RX, and XW contributed to the statistical analysis. DS and XW wrote the manuscript. All authors contributed to data collection and analysis. All authors contributed to the article and approved the submitted version.

## Funding

This work was supported by the Anhui Provincial Natural Science Foundation (1808085QH270; 2008085QH428), Fundamental Research Funds for the Central Universities (WK9110000121), and Key Research and Development Projects in Anhui Province (202004j07020017).

## Conflict of Interest

The authors declare that the research was conducted in the absence of any commercial or financial relationships that could be construed as a potential conflict of interest.
